# Whole exome sequencing (WES) on formalin-fixed, paraffin-embedded (FFPE) tumor tissue in gastrointestinal stromal tumors (GIST)

**DOI:** 10.1186/s12864-015-1982-6

**Published:** 2015-11-03

**Authors:** Annalisa Astolfi, Milena Urbini, Valentina Indio, Margherita Nannini, Chiara Giusy Genovese, Donatella Santini, Maristella Saponara, Anna Mandrioli, Giorgio Ercolani, Giovanni Brandi, Guido Biasco, Maria A. Pantaleo

**Affiliations:** “Giorgio Prodi” Cancer Research Center, University of Bologna, Bologna, Italy; Department of Specialized, Experimental and Diagnostic Medicine, S. Orsola-Malpighi Hospital, University of Bologna, Via Massarenti 9, 40138 Bologna, Italy; Pathology Unit, S. Orsola-Malpighi Hospital, University of Bologna, Bologna, Italy; Transplant, General and Emergency Surgery Department, S. Orsola-Malpighi Hospital, University of Bologna, Bologna, Italy

**Keywords:** Gastrointestinal stromal tumors (GIST), Formalin-fixed, paraffin-embedded (FFPE), Fresh frozen tissue, Next generation sequencing (NGS)

## Abstract

**Background:**

Next generation sequencing (NGS) technology has been rapidly introduced into basic and translational research in oncology, but the reduced availability of fresh frozen (FF) tumor tissues and the poor quality of DNA extracted from formalin-fixed, paraffin-embedded (FFPE) has significantly impaired this process in the field of solid tumors. To evaluate if data generated from FFPE material can be reliably produced and potentially used in routine clinical settings, we performed whole exome sequencing (WES) from tumor samples of Gastrointestinal stromal tumors (GIST), either extracted FF or FFPE, and from matched normal DNA.

**Methods:**

We performed whole exome enrichment and sequencing at 100bp in paired end on four GIST samples, either from FFPE or fresh-frozen tissue, and from matched normal DNA.

**Results:**

The integrity of DNA extracted from FFPE was evaluated by a modified RAPD PCR method, thus identifying high quality (HQ) and low quality (LQ) FFPE. DNA library production and exome capture was feasible for both classes of FFPE, despite the smaller yield and insert size of LQ-FFPE. WES produced data of equal quality from FF and FFPE, while only HQ-FFPE yielded an amount of data comparable to FF samples. Bioinformatic analysis showed that the percentage of variants called both in FF and FFPE samples was very high in HQ-FFPE, reaching 94-96 % of the total number of called variants. Classification of somatic variants by nucleotide substitution type showed that HQ-FFPE and FF had similar mutational profiles, while LQ-FFPE samples carried a much higher number of mutations than the FF counterpart, with a significant enrichment of C > T/G > A substitutions. Focusing on potential disease-related variants allowed the discovery of additional somatic variants in GIST samples, apart from the known oncogenic driver mutation, both from sequencing of FF and FFPE material. False positive and false negative calls were present almost exclusively in the analysis of FFPE of low quality. On the whole this study showed that WES is feasible also on FFPE specimens and that it is possible to easily select FFPE samples of high quality that yield sequencing results comparable to the FF counterpart.

**Conclusions:**

WES on FFPE material may represent an important and innovative source for GIST research and for other solid tumors, amenable of possible application in clinical practice.

**Electronic supplementary material:**

The online version of this article (doi:10.1186/s12864-015-1982-6) contains supplementary material, which is available to authorized users.

## Background

Massively parallel sequencing by next generation sequencing (NGS) technology has been rapidly introduced into basic and translational research in oncology, due to the ability of identifying the complete landscape of genetic alterations in many tumor types [1–5].

Most genotyping studies have been performed using fresh frozen (FF) tissues, and have provided great insights into the cancer molecular biology. However, the higher quality of DNA extracted from FF tissue is offset by the reduced availability of the samples, which does not allow to perform large-scale retrospective studies. Therefore in the recent years, many efforts have been addressed to set up strategies to apply massively parallel sequencing technology to formalin-fixed, paraffin-embedded (FFPE) specimens. While FFPE specimens are now frequently analyzed by amplicon-based or targeted-capture NGS panels [6–10], the possibility to reliably perform whole genome or whole exome sequencing (WES) in archival tumor samples still represents a challenge, both from the technical and bioinformatic point of view [11–16].

Gastrointestinal stromal tumors (GIST) are mesenchymal tumors that most frequently arise in the gastrointestinal tract. GIST are characterized by mutually exclusive KIT (85 %) or platelet-derived growth factor receptor alpha (PDGFRA) (5-10 %) gain of function mutations, leading to constitutive ligand-independent activation of receptor signalling [17–19]. The knowledge about the oncogenic mechanisms responsible for GIST onset paved the way for the effective introduction of tyrosine-kinase inhibitors (TKIs) in the standard treatment protocols and the recognition of the clinical impact and predictive significance of molecularly-defined subtypes [20, 21]. Up to now, about 10-15 % of GIST do not exhibit neither KIT or PDGFRA mutations and have been defined as KIT/PDGFRA wild type (WT), which represent an extremely heterogeneous subgroup, characterized by different subsets with distinct molecular hallmarks [22, 23].

In this complex scenario, in which the molecular biology plays a certain relevant role, but the FF specimens are often not available, the feasibility of high-throughput genomic studies on FFPE tissue would allow to perform larger prospective and retrospective studies on all these small subsets of GIST, expanding the reproducibility and the reliability of the data.

This study is aimed to develop a reliable approach to perform WES on archival tumor samples from GIST patients, in order to evaluate how data generated from FFPE material can be generated and potentially used in routine clinical settings. Herein we reported the first pivotal study on the comparison between data obtained by whole exome analysis on four FFPE and FF GIST samples, showing an high degree of concordance for all the variants found, including common polymorphism and novel somatic variants.

## Results and discussion

### FFPE-DNA integrity analysis

**Fig. 1:**
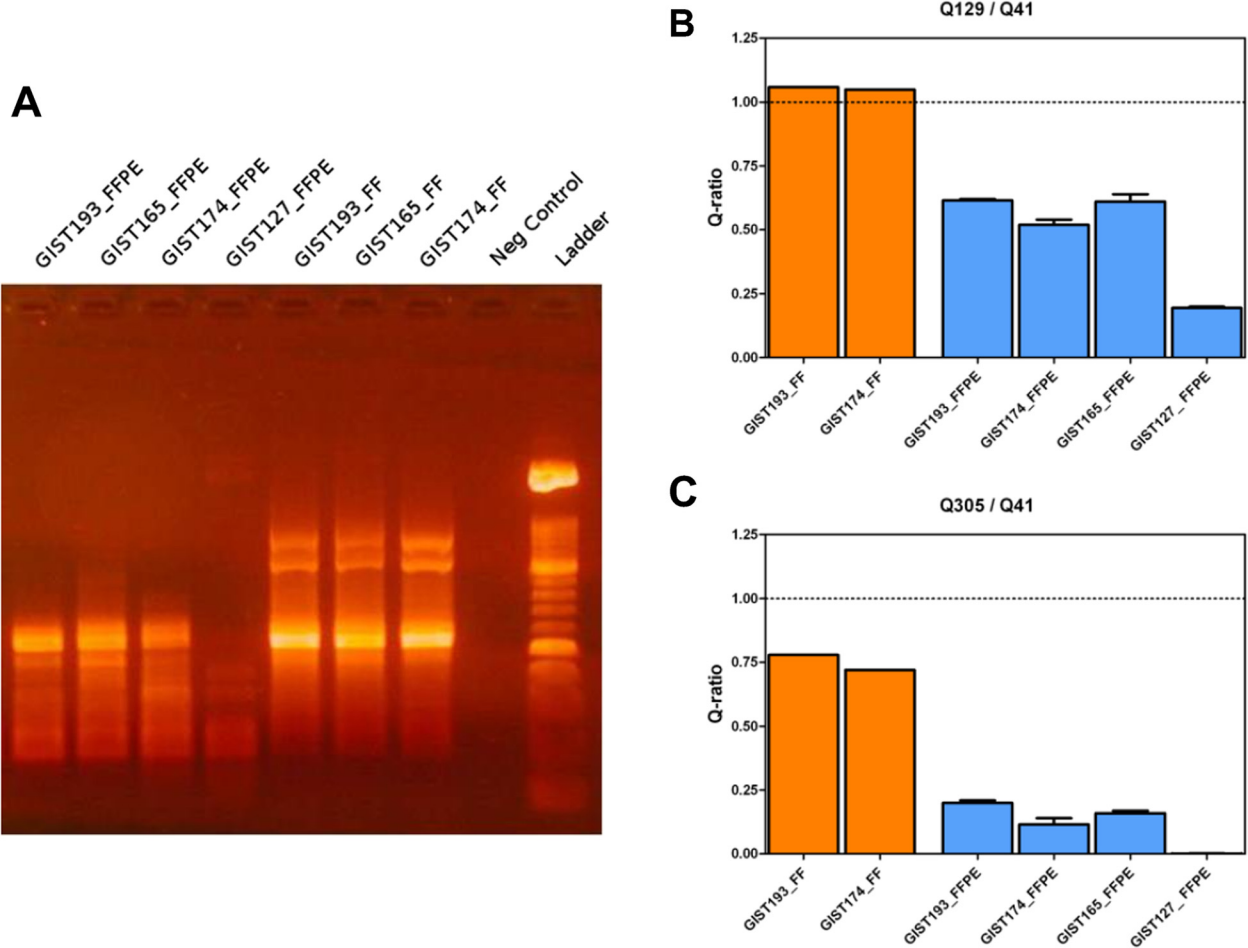
Quality control of DNA extracted from FFPE samples. **a** RAPD PCR performed on FF tumors and FFPE –derived DNA. **b-c**. KAPA HgDNA quantitative PCR QC kit, summarized as Q-score of the 129 bp vs the control 41 bp amplicon **(b)** and as the 305 bp vs the 41 bp **(c)**

To be able to produce clinically relevant and reliable data from FFPE samples it is necessary to quantify the degree of DNA degradation of FFPE-derived DNA. We used two different PCR-based methods, that produce a qualitative and quantitative assessment of DNA integrity. The first is a modification of the Random Amplified Polymorphic DNA assay (RAPD), that uses degenerated primers to amplify multiple fragments, of different length on the genome. DNA derived from FF samples amplifies a prevalent pool of fragments of around 500 bp, with multiple bands of higher size, while FFPE-derived DNA is either of high quality (HQ-FFPE) and shows the 500 bp band as the longest amplifiable fragment (GIST193, GIST174, GIST165), or of low quality (LQ-FFPE), thus showing only shorter amplified fragments of no more than 300–400 bp (GIST127) (Fig. 1a). These two categories of FFPE-derived DNA are identifiable in a larger panel of samples, where we detected HQ-FFPE DNA in approximately 40-50 % of samples (data not shown).

The other method is a quantitative-PCR that amplifies fragments of 41 bp, 129 bp and 305 bp of a highly conserved single copy gene. DNA quality is assessed by normalizing the concentration obtained with the 129 and 305 bp amplicon against the 41 bp amplicon (Q-ratio), where a Q-ratio of 1 represents intact DNA. This assay shows results in agreement with RAPD assay, where HQ-FFPE have a Q129/Q41 ratio above 0.5, and LQ-FFPE below 0.25 (Fig. 1b). Similarly, Q301/Q41 ratio of HQ-FFPE is a measurable value (>0.1), while in LQ-FFPE the amplification of the 301 bp fragment is almost undetectable (Q-ratio ≈ 0) (Fig. 1c).

### Exome enrichment and sequencing of FFPE samples

DNA library preparation and exome enrichment with Nextera Rapid Capture Exome Enrichment kit (Illumina) was then performed for three HQ-FFPE and one LQ-FFPE, for the paired FF tumor samples and normal counterpart from peripheral blood DNA. Tagmentation was performed on 100 ng of DNA, thus reaching an optimal library yield and size distribution for all the samples, except for the LQ-FFPE DNA (Additional file 1: Table S2). All the samples, including those derived from HQ-FFPE, yielded between 1.8 and 2.7 ug of DNA libraries, while the one derived from LQ-DNA reached the necessary 0.5 ug only with two independent reactions that were pooled. Average library size for FF- and PB-derived DNA was 295 ± 5 bp, while HQ-FFPE DNA yielded libraries of 230–240 bp, and LQ-FFPE did not exceed 190 bp (Additional file 1: Figure S1).

Libraries were then indexed, pooled and enriched for the exonic component, and then sequenced at 100 bp in paired – end. Lane-specific sequencing quality parameters (Density, % of clusters passing filter, % of bases ≥ Q30) were similar for FF and FFPE tumor samples, and for PB-derived DNA (Additional file 1: Table S3). In particular, the Average Q-score and the % of bases with Q-score ≥ Q30 were comparable for all the samples analyzed (Additional file 1: Table S4).

Despite similar performance of clustering and sequencing the LQ-FFPE sample (GIST127_FFPE) showed a much lower data yield (14 million reads), while HQ-FFPE produced the same amount of reads as FF samples (55 million reads for FF *vs* 56 million reads for HQ-FFPE samples) (Table 1). The percentage of PCR duplicates was approximately the same for all the samples, while the percentage of bases trimmed due to sequencing falling into adapters, primers and indexes was low for FF, PB and HQ-FFPE, while it was relevant in the GIST127_FFPE sample (20.7 vs 9.2 % on average). This result was expected, since DNA library dimension of the LQ-FFPE sample was below 200 bp. This is reflected also by the value of the average insert size, that is proportional to DNA integrity, with values proportionally decreasing from PB to FF, to HQ-FFPE and lastly to LQ-FFPE samples (Additional file 1: Figure S2).

**Table 1:** Sequencing quality and statistical parameters in FF, FFPE and PB samples

Patient	Sample	Total n° Reads	High Quality Bases (n°)	% Trimmed Bases	Unique Reads (n°)	% PCR duplicate	n° Mapped Reads	% Mapped Reads
GIST193	FF	63,523,138	5.84E + 09	8.1 %	58,598,506	7.8 %	57,879,604	98.8 %
	FFPE	54,852,570	4.97E + 09	9.4 %	49,208,829	10.3 %	48,499,561	98.6 %
	PB	42,361,296	3.80E + 09	10.3 %	39,964,111	5.7 %	39,385,105	98.6 %
GIST165	FF	53,644,770	4.95E + 09	7.7 %	49,587,098	7.6 %	49,019,552	98.9 %
	FFPE	50,570,884	4.59E + 09	9.3 %	45,332,397	10.4 %	44,682,820	98.6 %
	PB	42,235,292	3.87E + 09	8.3 %	39,957,754	5.4 %	39,460,107	98.8 %
GIST174	FF	56,267,604	5.10E + 09	9.3 %	52,033,110	7.5 %	51,338,819	98.7 %
	FFPE	64,625,134	5.87E + 09	9.2 %	58,169,850	10.0 %	57,529,198	98.9 %
	PB	52,136,904	4.69E + 09	10.0 %	48,822,001	6.4 %	48,110,730	98.5 %
GIST127	FF	48,223,180	4.42E + 09	8.4 %	44,804,080	7.1 %	44,294,468	98.9 %
	FFPE	14,203,932	1.14E + 09	20.7 %	12,496,962	12.0 %	12,310,812	98.1 %
	PB	43,078,472	3.86E + 09	10.4 %	40,476,585	6.0 %	39,924,959	98.4 %

Almost all the sequences after adaptor trimming and PCR duplicate removal mapped on the human genome hg19, since the percentage of mapped reads exceeded 98 % (Table 1).

Average coverage of the target exome region was comparable for FF- and FFPE-derived samples (FF: 55X - 71X; FFPE: 58X – 77X), except for the LQ-FFPE, that reached an average coverage of 17X (Table 2). Similarly, the percentage of the target region covered at least 10X was very high for both FF and FFPE-derived samples (92-96 %), while it was below 60 % for GIST127_FFPE (Table 2).

**Table 2:** Average coverage and percentage of target enriched region covered at least 1X and 10X

Patient	Sample	Average Coverage 37 Mb	% Nextera covered > = 1X	% Nextera covered > = 10X
GIST193	FF	71X	99.2 %	96.5 %
	FFPE	65X	99.2 %	95.8 %
	PB	43X	99.2 %	93.7 %
GIST165	FF	59X	99.2 %	95.0 %
	FFPE	58X	99.2 %	94.9 %
	PB	47X	99.2 %	93.6 %
GIST174	FF	61X	99.0 %	92.2 %
	FFPE	77X	99.0 %	94.3 %
	PB	52X	99.3 %	95.0 %
GIST127	FF	55X	99.0 %	92.9 %
	FFPE	17X	93.0 %	58.1 %
	PB	46X	99.0 %	92.2 %

Off-target sequencing is related to the amount of reads mapping outside the region targeted by the exonic capture, that is of 37 Mb with the Nextera Rapid Capture assay. The amount of off-target sequencing was from 39 to 53 %, the smaller value related to the LQ-FFPE sample (Additional file 1: Figure S3).

### Comparison of WES results of FFPE and FF samples

Sequencing data was analyzed with the pipeline described in the Methods section. First of all the degree of concordance between FFPE and Fresh samples was computed considering all the variants called, including common polymorphism and novel variants mapping on the 37 Mb Exome target region.

**Fig. 2:**
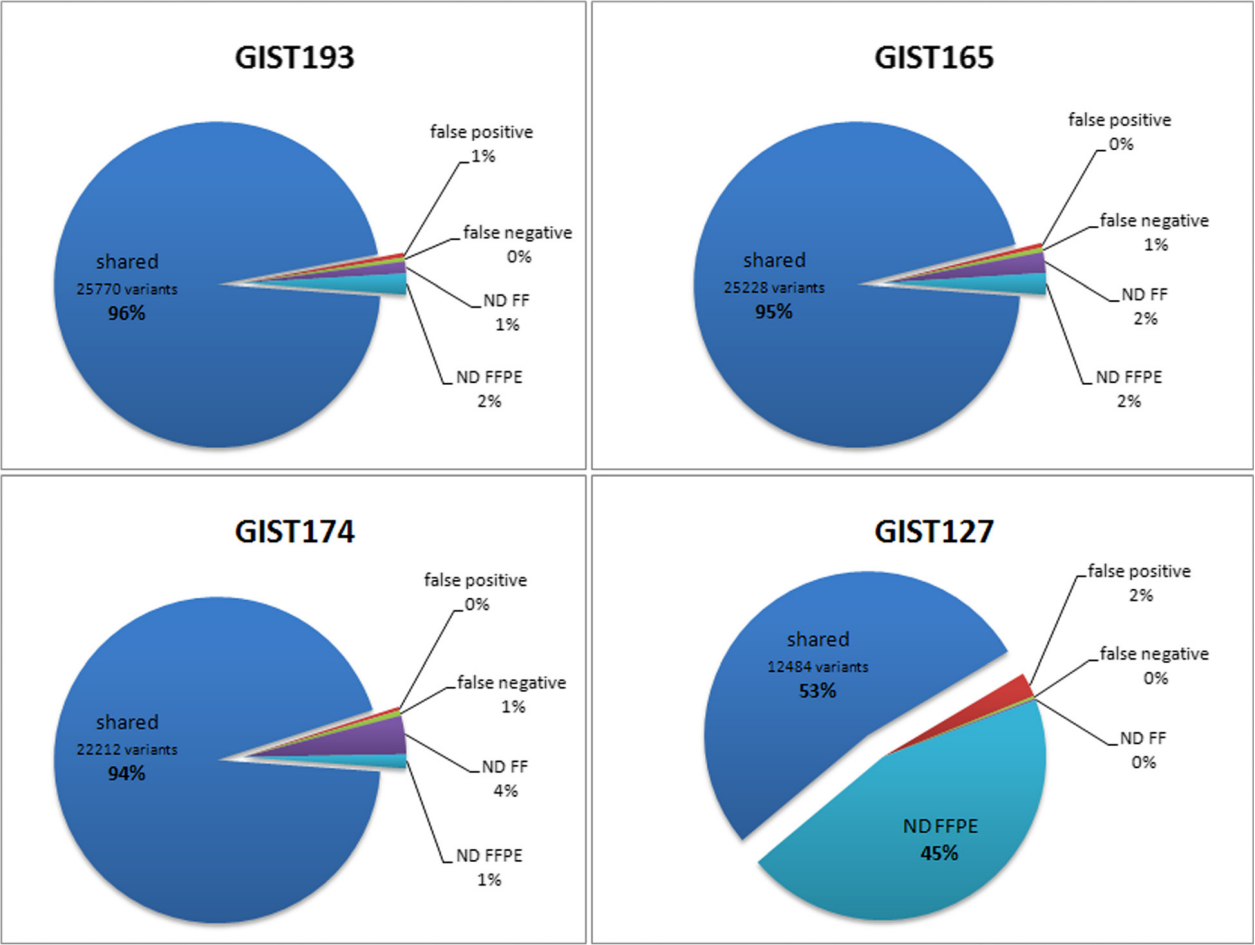
Concordance between FF and FFPE data. All the variants called, including common polymorphism and novel variants mapping on the 37 Mb Exome target region were classified as *Shared* if called in both FF and FFPE samples, as *False Negative* (FN) if called only in FF sample, as *False Positive* (FP) if detected only in FFPE, or ND if not sufficiently covered in either type of sample

As shown in Fig. 2 the percentage of shared variants, called both in FF and FFPE samples, is very high in HQ-FFPE, reaching 94-96 % of the total number of called variants. Conversely, in GIST127 sample the FFPE samples loses almost half of the variants called in the FF sample, since the number of variants not determined in FFPE is up to 45 % of the total number.

**Fig. 3:**
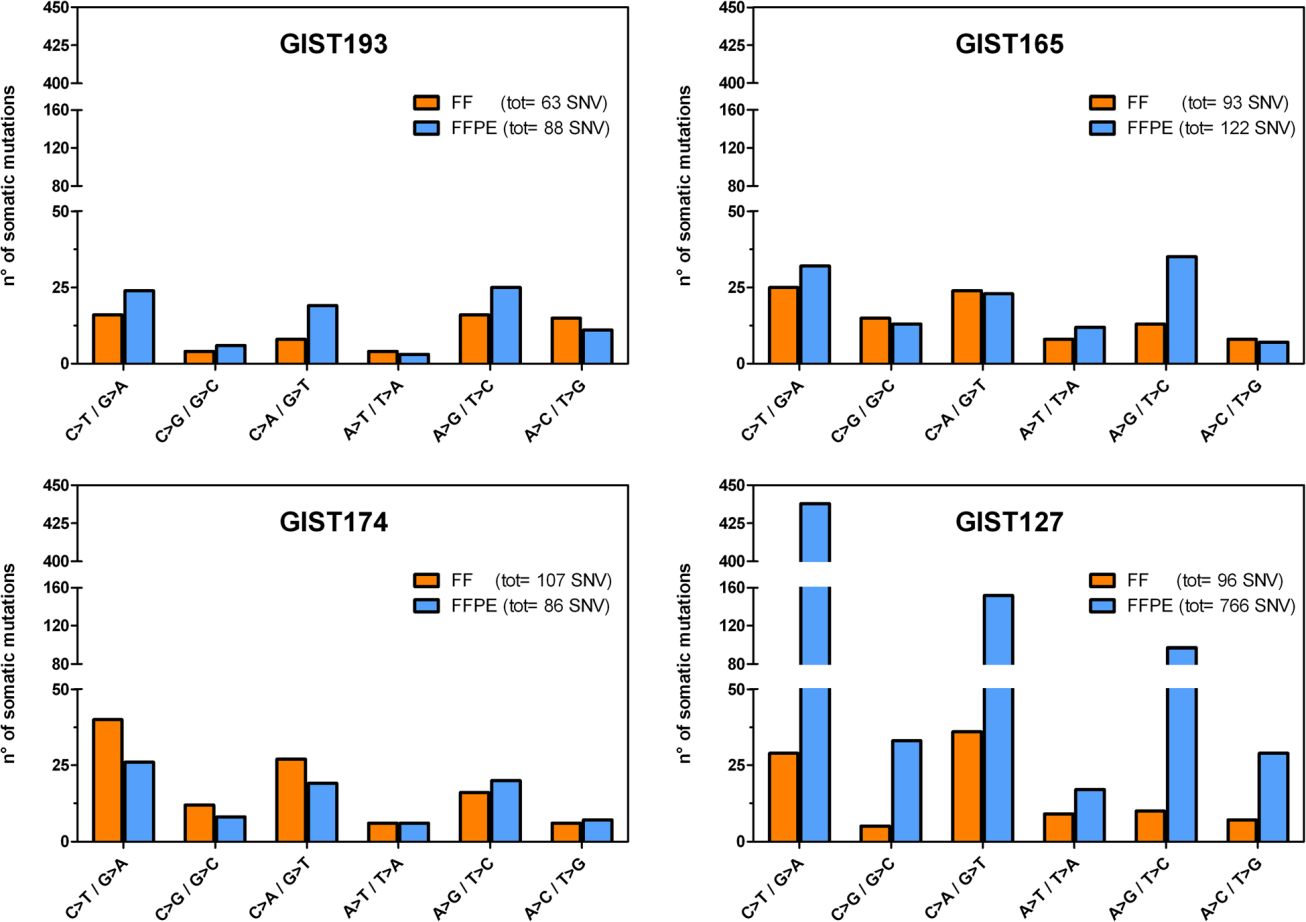
Total number of somatic SNVs detected in FF and FFPE samples, stratified based on the type of nucleotide substitution

We selected only the somatic single nucleotide variants (SNVs) and then classified them based on the type of nucleotide substitution (Fig. 3). In all the HQ-FFPE the number of mutations falling in the different classes are comparable to the corresponding FF sample while in the GIST127 FFPE the total number of putative somatic SNVs is higher than the corresponding FF sample and in particular the most enriched class of mutation is the C > T/G > A as expected by cytosine deamination due to formalin fixation.

To identify the disease-related variants, we further restricted the dataset to the somatic non-synonimous (missense, nonsense and stop loss) SNVs and InDels located in the coding region and splicing sites.

**Fig. 4:**
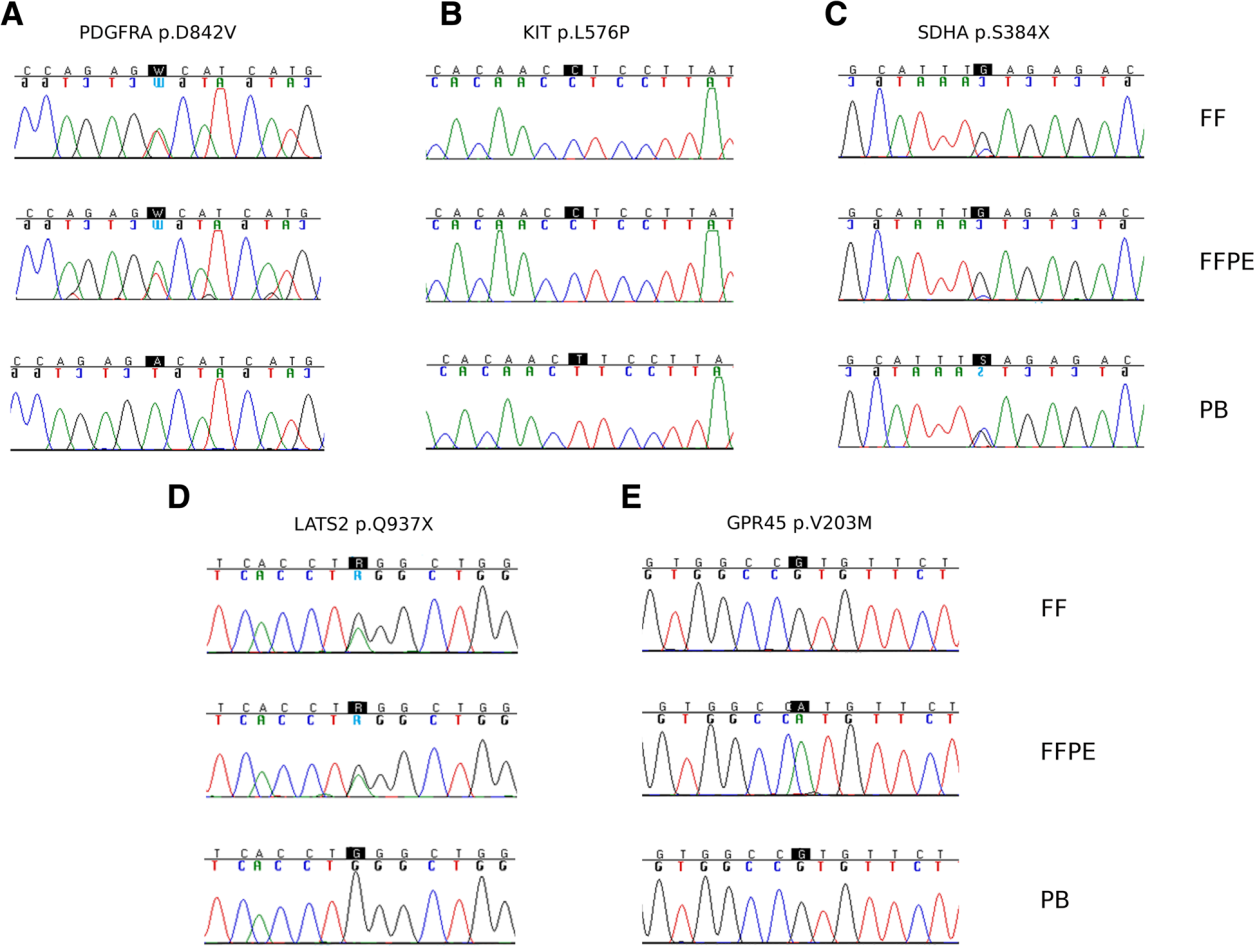
Validation of selected somatic mutations by Sanger sequencing on FF and FFPE tumor DNA and on PB-derived DNA. **a** PDGFRA p.D842V mutation detected in GIST165 tumor DNA from FF and FFPE samples. **b** KIT p.L576P mutation present in GIST174 patient, both in FF and FFPE. **c** SDHA p.S384X detected in patient GIST193 in heterozygosis in the germline, and in homozygosis in tumor DNA (both from FF and FFPE). **d** *False negative* LATS2 p.Q937X somatic stop-gain mutation. This mutation is present in both FF and FFPE samples, but fails to be detected by WES of FFPE due to low coverage. **e** *False positive* GPR45 p.V203M missense variant. This putative mutation is a present only in FFPE and not in FF sample from GIST127, probably due to cytosine deamination induced by formalin fixation

By this we highlighted 4, 13, 27 and 26 somatic mutations in the four FF samples (GIST193, GIST165, GIST174 and GIST127, respectively, Additional file 1: Table S5). These mutations were defined as *Shared Variant*s if present also in FFPE (3, 13, 24 and 13, respectively). We confirmed the presence of known KIT and PDGFRA mutations also in FFPE samples (Table 3, Fig. 4a, b), and also the presence of truncating SDHA mutation in GIST193, even if not reported in the list of somatic variants, being a germinal mutation with LOH in the tumor sample (Fig. 4c). Overall WES, both performed on FF and FFPE samples, allowed the discovery of other somatic variants in GIST samples, apart from the known oncogenic driver mutations. It is worth noting that the number of mutations carried by SDHA-driven tumors is almost negligible with respect all other GIST molecular subgroups; moreover, WES analysis proved to be informative even for tumors driven by well-known molecular alterations, being able to identify clinically relevant mutations, as in the case of PTEN R233X mutation in GIST174, evidenced both in FF and FFPE sample (Additional file 1: Table S5).

**Table 3:** Detection of known pathogenic mutations carried by the GIST samples analyzed as evidenced by exome sequencing in FF and FFPE samples

Patient	Chr:Position	GENE	cDNA	PROTEIN	FF Ref_Cov/Alt_Cov (Ratio)	FFPE Ref_Cov/Alt_Cov (Ratio)	PB Ref_Cov/Alt_Cov (Ratio)
GIST174	4:55593661	KIT	c.T1727C	p.L576P	2/160 (98.8 %)	12/236 (95.2 %)	116/0 (0 %)
GIST165	4:55152093	PDGFRA	c.A2525T	p.D842V	109/35 (24.3 %)	57/32 (36.0 %)	110/0 (0 %)
GIST193	5:235345	SDHA	c.C1151G	p.S384X	4/50 (92.6 %)	5/38 (88.4 %)	23/24 (51.1 %)

*Ref* reference, *Alt* alternative, *Cov* Coverage

*False Negative* variants, meaning those not called in the FFPE sample, were very few in HQ-FFPE (0–3 per sample), while very frequent in LQ-FFPE sample (13/26 variants identified in the FF sample). Anyway *False Negative* calls were mostly due to low coverage in FFPE samples (Fig. 4d). Conversely *False Positive* variants, i.e., present only in the FFPE samples, were negligible in HQ-FFPE (only one FP in each sample), but are particularly enriched in LQ-FFPE where they represented more than 60 % of the variants identified in FFPE (20 out of 33, Additional file 1: Table S5). Most of the false positive calls in the low quality FFPE were C > T and G > A substitutions (75 %). These events were real artifacts introduced by formalin fixation and not sequencing errors, since they were confirmed by Sanger sequencing (Fig. 4e).

It is well known that GIST represent an heterogeneous set of different clinical and biological entities, each of which characterized by a unique molecular profile, highlighting the relevant role of molecular biology in this disease, both in research settings and in clinical practice. In this contest, massively parallel sequencing emerged as a promising tool, allowing a complete picture of genetic alterations in many tumor types, including GIST [23, 24]. However, up to now this technology has been severely limited by the lack of FF tissue banks, needful for conducting large-scale studies, which are required in a rare tumor as GIST, in order to obtain reliable and transferable data into clinical practice.

Some evidences have been already reported on the feasibility of genome sequencing on FFPE specimens, representing the widest archive of tumor samples [11–16]. Herein we reported the first pivotal study on the comparison between data obtained by whole exome analysis on four FFPE and FF GIST samples, showing an high degree of concordance for all the variants found, including common polymorphism and novel somatic variants.

Indeed we know that targeted sequencing of clinically relevant mutation panels has become a feasible approach, that is increasingly applied in clinical practice to aid diagnosis and treatment choice [10, 25]. However there are many advantages to WES over targeted NGS approaches: first of all, given that the list of clinically actionable or informative mutations is increasing, the targeted panels become progressively less beneficial for clinical application; at the same time clinical WES is slowly turning into a rapid, cost-efficient, and straightforward technique, that can be amenable to routine application in clinical settings. Thus, given that the number of informative cancer mutations is rising, the application of WES analysis on archived tumor samples will definitely become an urgent need. Therefore, our results demonstrate that this analysis is not only feasible on at least a significant proportion of FFPE tumor samples, but also that the results are reliable, and almost superimposable to FF samples.

There are very few other reports on this topic; in particular, Hedegaard et al. achieved only partially positive results, with a poor percentage of successful exome library preparation and sequencing from FFPE (29 %) and an overly high representation of PCR duplicates (30 %), highlighting some reproducible issues of WES sequencing from FFPE, as the shorter insert sizes and the presence of longer adapter sequences in the reads [15]. Our approach has solved these technical issues, since we were able to produce DNA libraries from all the FFPE samples, and keep the PCR duplicates at levels comparable to FF samples.

Conversely, Kerick et al. and Van Allen et al., both using a different approach than ours for exome library prep (Agilent SureSelect), have reported an high reproducibility of SNV detection between FF and FFPE, only slightly lower than ours, but both authors did not take into account of clinically relevant somatic mutations and did not correlate the sequencing performance with FFPE quality [11, 16]. Here we show that not only it is possible to achieve results comparable to FF when performing exome sequencing on FFPE tumor samples, but also that there are ways to score the quality of FFPE DNA that predict the reliability of sequencing results, with a degree of concordance with FF sample that is over 94 %. Actually, as the predominant errors introduced by WES on FFPE are the false positives due to cytosine deamination (C > T and G > A substitutions), that can represent up to 60 % of the called variants in FFPE samples, we also show that by selecting only high quality FFPE this bias becomes negligible. In fact we also showed that low quality FFPE carries a higher mutational burden, that is explained in part by cytosine deamination and also by the overall extent of DNA damage, thus explaining why all categories of base substitutions are enriched in LQ-FFPE, anyway with a marked predominance of transitions (10X – 15X) versus transversions (2X – 6X).

The study results have important implications in GIST’s translational research. The high degree of concordance of the data would allow to expand the NGS analysis to all archived GIST specimens, thus enlarging the sample size analyzed. In a rare disease as GIST, where it is methodologically difficult designing prospective studies due to their very low incidence, the possibility to open up the analysis to the all archived specimens, may offer the opportunity to perform larger retrospective studies, using innovative technologies, without the limit of availability of FF tumor tissue. Moreover, it may also allow applying these more recent tools on the wide case series of the historical conducted clinical trials, that represent the main available source of well-selected GIST patients, providing novel and more reliable interpretations of these historic data [26, 27]. Finally, the possibility to perform genomic studies on a large number of samples would also improve the knowledge on the biological background of many kinds of tumor, including GIST, allowing to better define the real prognostic and predictive value of several biological markers, not yet transferred into clinical practice because of their role still uncertain. This aspect is even more relevant for rare cancers, as GIST, for which it is extremely difficult to make studies with prognostic and predictive purpose, for the need of an adequate number of patients to analyze and follow for a long time.

## Conclusions

Given the preliminary but promising results above-reported, it is mandatory to confirm and validate all technical steps, and all analytical processes, raising the challenge of optimize and transfer this assay into clinical practice [27]. Firstly, robust DNA extraction and sequencing library construction protocols are required. Secondly, analytical protocols that can be applicable on limited amounts of tissue and extracted DNA are mandatory especially when the samples available for testing come from small-core needle biopsies and fine-needle aspirations. Finally, the analytical process should be standardized, and validated in order to let this technology feasible for a clinical use.

In conclusion, WES on FFPE specimens may represent an important and innovative source for GIST research, and further investigations are required in order to better assess the assay.

## Methods

### Sample collection

This study was approved by the institutional review board of Azienda Ospedaliero-Universitaria Policlinico S. Orsola-Malpighi, Bologna, Italy (approval number 113/2008/U/Tess). All patients provided written informed consent. Tumor specimens were collected during surgery and reviewed by the pathologist who cut a portion to be snap-frozen and conserved in liquid nitrogen (FF). The tumor specimen was then fixed in 10 % NBF (Formalin Solution, Neutral Buffered) for no less than 6 h and not more than 72 h, then dehydrated and included in paraffin (FFPE). For DNA extraction, at least 2–3 slices of 10 um of thickness were cut from the paraffin block and superimposed to a 3um H&E-stained slide. Clinical and biological data of the patients included in the study are listed in Additional file 1: Table S1.

### DNA extraction

Manual macrodissection of the tumoral area, identified by superimposition of a H&E stained glass, was performed on the FFPE slide using a scalpel. Macrodissected FFPE and fresh frozen tissues were digested over-night at 56 °C in ATL buffer with the addition of proteinase K (Qiagen). DNA extraction was then continued with QIAamp DNA micro kit (Qiagen). DNA from peripheral blood was extracted with QIAamp DNA mini kit following manufacturer’s instructions. DNA concentration was determined with both spectrophotometric (Nanodrop) and fluorometric (Picogreen dsDNA kit, Life Technologies) methods. On average, the concentration measured with picogreen was half the concentration estimated with Nanodrop.

### FFPE quality check

#### RAPD-modified method

This assay was taken from the RAPD method described by (3) and modified as follows: 2.5 – 5 ng of DNA from FFPE or FF DNA were amplified with 0.5 μM RAPD primers (FW: 5'-aatcgggctg-3; REV: 5‘-gaaacgggtg-3') with 0.5 U of KAPA 2G FAST HotStart TAQ polymerase (Kapa Biosystems), 2 mM MgCl2, 200 μM dNTPs. PCR was perfomed for 40 cycles (95 °C, 15 s; 37 °C, 15 s; 72 °C, 1 min).

#### KAPA HgDNA quantification and QC kit

This assay is based on quantitative-PCR amplification of a 41 bp, 129 bp and 305 bp fragment of a highly conserved single copy gene (KAPA Human Genomic DNA Quantification and QC Kit, Kapa Biosystems). 5 ng of DNA were amplified with KAPA SybrFast qPCR Master Mix on a ABI Prism 7900 HT (Applied Biosystems) following manufacturer’s instructions. The 41 bp amplicon is used for absolute quantification of DNA samples against a set of DNA standards. DNA quality is assessed by normalizing the concentration obtained with the 129 and 305 bp amplicon against the one obtained with the 41 bp assay (Q-score). Theoretically integer DNA has an optimal Q-score ≈ 1 for both the Q129/Q41 and the Q305/Q41 ratios.

### Next Generation sequencing

WES was performed on DNA isolated from fresh frozen and FFPE tumor tissue and from matched normal peripheral blood DNA. Whole exome libraries were prepared in accordance with Nextera Rapid Capture Exome Enrichment protocol (Illumina). Briefly 100 ng of genomic DNA was tagmented (tagged and fragmented) by the Nextera transposome technique to an average library size of 290 bp (190–230 bp for the FFPE-derived tumor samples). DNA Library dimension were measured with DNA 1000 chip on the Agilent 2100 Bioanalyzer workstation (Agilent). DNA libraries were then pooled, denatured to single stranded DNA and hybridized to biotin-labeled 80-mer probes designed to enrich 214,126 targeted exonic regions, then eluted from magnetic beads. 

Exome enriched DNA libraries were quality-checked and sized with Agilent DNA 1000 or 7500 chips on the Bioanalyzer 2100 (Agilent Technologies,Taiwan), then quantified using a fluorometric assay (QuantIT Picogreen assay, Life Technologies). 12pM paired-end libraries were amplified and ligated to the flowcell by bridge PCR, and sequenced at 2x100bp read length, using Illumina Sequencing by synthesis (SBS) technology. An average of 55 million reads for FF and of 46 million reads for FFPE samples were obtained for WES analysis.

### Bioinformatic analysis

Adapter removal and quality trimming were performed with the tool AdapterRemoval using the default parameters except the threshold for trimming low quality bases (Q < 10), meaning that consecutive stretches of bases from both the 5' and 3' end of the reads with Phred Quality of 10 or lower were trimmed, and the minimum read length set to 30 nucleotides.

After trimming, the short reads were mapped on the human reference genome hg19 with BWA software. The alignments were processed with samtools to remove PCR duplicate and with GATK in order to perform local realignment around the indel position, base quality score recalibration and insertion/deletion calling (InDels), while variation calling was performed with MuTect thus identifying all the point mutations in the sample (SNVs). Variants were considered for the further analysis if mapping on the 37 Mb Nextera target region, while the remaining were defined as “off-target” and excluded. This set of variants (SNVs and the InDels called with Mutect and GATK respectively) detected in both Fresh and FFPE samples was reduced in order to keep into account only the variant that are defined as “high-quality” at least in one of the two samples according the following criteria met in Fresh sample, FFPE sample or both: MuTect/GATK filter label = KEEP/PASS, Depth of coverage > 10, Ratio of alternate allele > = 0.2. The coverage of alternate and reference alleles were re-counted with the function *mpileup* of samtools and no upper limits in the depth of coverage was set.

The resulting dataset was considered to estimate the agreement between FFPE and Fresh data. We defined:

- If depth of coverage < = 10, Undetermined variants (ND);
- If depth of coverage >10:
  - O Shared variants, if variant is called in both FFPE and Fresh samples;
  - O FFPE False positive, if variant is called in FFPE and absent in Fresh sample;
  - O FFPE False negative, if variant is called in Fresh and absent on FFPE sample.

To identify the tumor–related events, variants present in dbSNP and 1000Genomes with frequency greater than 1 % were excluded. Thus all the variants either not reported, or present in dbSNP but with a frequency lower that 1 % or with no frequency reported were retained in the analysis. All variants from the matched normal-tumor pairs that were unique in the tumor sample were called as Somatic (using Samtools *mpileup* funtion). To call the somatic variants we relaxed the depth of coverage threshold to > = 6X to take into account the higher multiplexing of PB samples, the lower average coverage of poor quality FFPE, and the need to increase the sensitivity of the assay at the lower limit of detection. The whole set of somatic variants was manually checked within the BAM file in order to exclude alignment errors that in most cases occur in repetitive regions. The effect of coding SNV was predicted at the protein level with a suite of computational tools, such as SIFT and PROVEAN. Truncations and frameshift mutations were analyzed in relation to the annotations available on the protein sequence (e.g., from UniProt, PFAM, SCOP) in order to identify possible domain/site loss, disruption or gain that can affect protein function.

### Sanger sequencing

Validation of selected somatic variants was performed on DNA extracted from FF and FFPE tumor samples and from PB as a source of germline DNA. The genomic region surrounding the putative mutation was amplified with Polymerase Chain Reaction (PCR) using specific primer pairs designed with Primer Express 3.0 Software (Applied Biosystem). PCR products were then purified with the Qiaquick PCR purification kit (Qiagen, Milan, Italy) and sequenced on both strands using the Big Dye Terminator v1.1 Cycle Sequencing kit (Applied Biosystems). Sanger sequencing was performed on ABI 3730 Genetic Analyzer (Applied Biosystems).

## Additional file

Additional file 1:
**Table S1.** Clinical and biological data of the patients included in the study. **Table S2.** Tagmented DNA library and post-enrichment pooled library yield and average size. **Table S3.** Lane-specific sequencing quality parameters (cluster density, percentage of clusters passing filter, total number or reads passing filter and percentage of bases with quality ≥ Q30). **Table S4.** Sample-specific sequencing quality scores (percentage of bases with quality ≥ Q30 and average Q-score). **Table S5.** List of somatic somatic disease-related mutations (non-synonimous and nonsense SNVs and InDels located in the coding region and splicing sites). Variants are flagged as shared if present both in FF and FFPE samples, FP if called only in FFPE and FN if called only in FF. Ref: reference; Alt: alternative; Cov: Coverage. **Figure S1.** Tagmented DNA library size distribution as analyzed by Agilent DNA 1000 kit. **Figure S2.** Average insert size of the exome libraries sequenced at 100bp x 2. **Figure S3.** Percentage of reads mapping on-target and off-target, i.e., outside the 37Mb Nextera target region used for exome enrichment. (DOCX 100 kb)

## Abbreviations

WES: Whole exome sequencing
FFPE: Formalin-fixed, paraffin-embedded
GIST: Gastrointestinal stromal tumors
FF: Fresh frozen tissue
NGS: Next generation sequencing
HQ: High quality
LQ: Low quality
PDGFRA: Platelet-derived growth factor receptor alpha
WT: Wild type
RAPD: Random Amplified Polymorphic DNA assay
SNVs: Single nucleotide variants
SBS: Sequencing by synthesis

## References

[1] Mardis ER (2011). A decade’s perspective on DNA sequencing technology. Nature.

[2] Shendure J, Ji H (2008). Next-generation DNA sequencing. Nat Biotechnol.

[3] Campbell PJ, Stephens PJ, Pleasance ED, O'Meara S, Li H, Santarius T (2008). Identification of somatically acquired rearrangements in cancer using genome-wide massively parallel paired-end sequencing. Nat Genet..

[4] Shah SP, Köbel M, Senz J, Morin RD, Clarke BA, Wiegand KC (2009). Mutation of FOXL2 in granulosa-cell tumors of the ovary. N Engl J Med..

[5] Mardis ER, Ding L, Dooling DJ, Larson DE, McLellan MD, Chen K (2009). Recurring mutations found by sequencing an acute myeloid leukemia genome. N Engl J Med..

[6] Hadd AG, Houghton J, Choudhary A, Sah S, Chen L, Marko AC (2013). Targeted, high-depth, next-generation sequencing of cancer genes in formalin-fixed, paraffin-embedded and fine-needle aspiration tumor specimens. J Mol Diagn..

[7] Zhang L, Chen L, Sah S, Latham GJ, Patel R, Song Q (2014). Profiling cancer gene mutations in clinical formalin-fixed, paraffin-embedded colorectal tumor specimens using targeted next-generation sequencing. Oncologist..

[8] Spencer DH, Sehn JK, Abel HJ, Watson MA, Pfeifer JD, Duncavage EJ (2013). Comparison of clinical targeted next-generation sequence data from formalin-fixed and fresh-frozen tissue specimens. J Mol Diagn..

[9] Wong SQ, Li J, Tan AY, Vedururu R, Pang JM, Do H (2014). Sequence artefacts in a prospective series of formalin-fixed tumours tested for mutations in hotspot regions by massively parallel sequencing. BMC Med Genomics.

[10] Frampton GM, Fichtenholtz A, Otto GA, Wang K, Downing SR, He J (2013). Development and validation of a clinical cancer genomic profiling test based on massively parallel DNA sequencing. Nat Biotechnol.

[11] Kerick M, Isau M, Timmermann B, Sültmann H, Herwig R, Krobitsch S (2011). Targeted high throughput sequencing in clinical cancer settings: formaldehyde fixed-paraffin embedded (FFPE) tumor tissues, input amount and tumor heterogeneity. BMC Med Genomics.

[12] Yost SE, Smith EN, Schwab RB, Bao L, Jung H, Wang X (2012). Identification of high-confidence somatic mutations in whole genome sequence of formalin-fixed breast cancer specimens. Nucleic Acids Res.

[13] Li P, Conley A, Zhang H, Kim HL (2014). Whole-Transcriptome profiling of formalin-fixed, paraffin-embedded renal cell carcinoma by RNA-seq. BMC Genomics.

[14] Wong SQ, Li J, Salemi R, Sheppard KE, Do H, Tothill RW (2013). Targeted-capture massively-parallel sequencing enables robust detection of clinically informative mutations from formalin-fixed tumours. Sci Rep..

[15] Hedegaard J, Thorsen K, Lund MK, Hein AM, Hamilton-Dutoit SJ, Vang S (2014). Next-generation sequencing of RNA and DNA isolated from paired fresh-frozen and formalin-fixed paraffin-embedded samples of human cancer and normal tissue. PLoS One.

[16] Van Allen EM, Wagle N, Stojanov P, Perrin DL, Cibulskis K, Marlow S (2014). Whole-exome sequencing and clinical interpretation of formalin-fixed, paraffin-embedded tumor samples to guide precision cancer medicine. Nat Med..

[17] Corless CL, Fletcher JA, Heinrich MC (2004). Biology of gastrointestinal stromal tumors. J Clin Oncol..

[18] Hirota S, Isozaki K, Moriyama Y, Hashimoto K, Nishida T, Ishiguro S (1998). Gain of function mutations of c-kit in human gastrointestinal stromal tumors. Science..

[19] Heinrich MC, Corless CL, Duensing A, McGreevey L, Chen CJ, Joseph N (2003). PDGFRA activating mutations in gastrointestinal stromal tumors. Science..

[20] Heinrich MC, Corless CL, Demetri GD, Blanke CD, von Mehren M, Joensuu H (2003). Kinase mutations and imatinib response in patients with metastatic gastrointestinal stromal tumor. J Clin Oncol..

[21] Heinrich MC, Maki RG, Corless CL, Antonescu CR, Harlow A, Griffith D (2008). Primary and secondary kinase genotypes correlate with the biological and clinical activity of sunitinib in imatinib-resistant gastrointestinal stromal tumor. J Clin Oncol..

[22] Nannini M, Biasco G, Astolfi A, Pantaleo MA (2013). An overview on molecular biology of KIT/PDGFRA wild type (WT) gastrointestinal stromal tumours (GIST). J Med Genet..

[23] Nannini M, Astolfi A, Urbini M, Indio V, Santini D, Heinrich MC (2014). Integrated genomic study of quadruple-WT GIST (KIT/PDGFRA/SDH/RAS pathway wild-type GIST). BMC Cancer..

[24] Pantaleo MA, Astolfi A, Indio V, Moore R, Thiessen N, Heinrich MC (2011). SDHA loss-of-function mutations in KIT-PDGFRA wild-type gastrointestinal stromal tumors identified by massively parallel sequencing. J Natl Cancer Inst..

[25] Jones S, Anagnostou V, Lytle K, Parpart-Li S, Nesselbush M, Riley DR (2015). Personalized genomic analyses for cancer mutation discovery and interpretation. Sci Transl Med.

[26] Demetri GD, von Mehren M, Blanke CD, Van den Abbeele AD, Eisenberg B, Roberts PJ (2002). Efficacy and safety of imatinib mesylate in advanced gastrointestinal stromal tumors. N Engl J Med..

[27] Demetri GD, van Oosterom AT, Garrett CR, Blackstein ME, Shah MH, Verweij J (2006). Efficacy and safety of sunitinib in patients with advanced gastrointestinal stromal tumour after failure of imatinib: a randomised controlled trial. Lancet.

