# Supplementary material for: Whole exome sequencing (WES) on formalin-fixed, paraffin-embedded (FFPE) tumor tissue in gastrointestinal stromal tumors (GIST)
Source: BMC Genomics. 2015 Nov 3;16:892. doi: 10.1186/s12864-015-1982-6 (PMC4630927; doi:10.1186/s12864-015-1982-6)
Supplement: Additional file 1: — Table S1. Clinical and biological data of the patients included in the study. Table S2. Tagmented DNA library and post-enrichment pooled library yield and average size. Table S3. Lane-specific sequencing quality parameters (cluster density, percentage of clusters passing filter, total number or reads passing filter and percentage of bases with quality ≥ Q30). Table S4. Sample-specific sequencing quality scores (percentage of bases with quality ≥ Q30 and average Q-score). Table S5. List of somatic somatic disease-related mutations (non-synonimous and nonsense SNVs and InDels located in the coding region and splicing sites). Variants are flagged as shared if present both in FF and FFPE samples, FP if called only in FFPE and FN if called only in FF. Ref: reference; Alt: alternative; Cov: Coverage. Figure S1. Tagmented DNA library size distribution as analyzed by Agilent DNA 1000 kit. Figure S2. Average insert size of the exome libraries sequenced at 100bp x 2. Figure S3. Percentage of reads mapping on-target and off-target, i.e., outside the 37Mb Nextera target region used for exome enrichment. (DOCX 100 kb) [file 12864_2015_1982_MOESM1_ESM.docx]

**Additional file 1**

**Table S1.** Clinical and biological data of the patients included in the study.

| **ID** | **Sex** | **Age** | **Site** | **Disease status at diagnosis** | ***KIT/PDGFRA/SDH* mutational status** |
| --- | --- | --- | --- | --- | --- |
| **GIST127** | F | 63 | Ileum | Localized | WT |
| **GIST174** | M | 59 | Stomach | Metastatic | KIT exon 11 p.L576P |
| **GIST165** | M | 50 | Stomach | Metastatic | PDGFRA exon 18 p.D842V |
| **GIST193** | F | 31 | Stomach | Localized | SDHA exon 9 p.S384X |

**Table S2**. Tagmented DNA library and post-enrichment pooled library yield and average size.

|  | **Tagmented Library yield** | **Tagmented library dimension (bp)** | **Post enrichment pooled library yield** | **Post enrichment library dimension** |
| --- | --- | --- | --- | --- |
| **GIST193_FF** | 1.8 ug (72.8 ng/ul) | 290 | 20.7 ng/ul  (98.6 nM) | 323 bp |
| **GIST165_FF** | 2.7 ug (106 ng/ul) | 294 |  |  |
| **GIST174_FF** | 2.1 ug (85.0 ng/ul) | 274 |  |  |
| **GIST193_FFPE** | 2.4 ug (94.5 ng/ul) | 237 | 12.3 ng/ul  (63.8 nM) | 297 bp |
| **GIST165_FFPE** | 2.7 ug (106 ng/ul) | 230 |  |  |
| **GIST174_FFPE** | 2.5 ug (100 ng/ul) | 240 |  |  |
| **GIST127_FF** | 2.3 ug (91.5 ng/ul) | 290 | 17.8 ng/ul  (69.1 nM) | 395 bp |
| **GIST127_FFPE (1)** | 0.36 ug (14.5 ng/ul) | 183 |  |  |
| **GIST127_FFPE (2)** | 0.20 ug (7.9 ng/ul) | 190 |  |  |
| **GIST193_PB** | 2.1 ug (83.1 ng/ul) | 291 | 32.7 ng/ul  (139 nM) | 361 bp |
| **GIST165_PB** | 1.9 ug (77.1 ng/ul) | 320 |  |  |
| **GIST174_PB** | 2.1 ug (82.7 ng/ul) | 312 |  |  |
| **GIST127_PB** | 2.1 ug (82.8 ng/ul) | 295 |  |  |

**Table S3.** Lane-specific sequencing quality parameters (cluster density, percentage of clusters passing filter, total number or reads passing filter and percentage of bases with quality ≥ Q30)

| **Lane** | **Sample** | **index** | **Density (K/mm2)** | **% clusters PF** | **Reads PF** | **% ≥ Q30** |
| --- | --- | --- | --- | --- | --- | --- |
| 1 | **GIST193_FF** | N702 | 746 ± 74 | 85.7 % | 89.1 M | 84.0 % |
|  | **GIST165_FF** | N704 |  |  |  |  |
|  | **GIST174_FF** | N705 |  |  |  |  |
|  | | | | | | |
| 2 | **GIST193_FFPE** | N702 | 723 ± 80 | 86.7 % | 87.4 M | 85.1 % |
|  | **GIST165_FFPE** | N704 |  |  |  |  |
|  | **GIST174_FFPE** | N705 |  |  |  |  |
|  | | | | | | |
| 3 | **GIST165_PB** | N702 | 757 ± 83 | 84.7 % | 89.4 M | 83.5 % |
|  | **GIST174_PB** | N704 |  |  |  |  |
|  | **GIST127_PB** | N705 |  |  |  |  |
|  | **GIST193_PB** | N706 |  |  |  |  |
|  | | | | | | |
| 4 | **GIST127_FF** | N702 | 779 ± 64 | 86.4 % | 94.0 M | 82.2 % |
|  | **GIST127_FFPE** | N705 |  |  |  |  |
|  | ---------- | N704 |  |  |  |  |

**Table S4.** Sample-specific sequencing quality scores (percentage of bases with quality ≥ Q30 and average Q-score)

| **Patient** | **Sample** | **%Bases > Q30** | **Average Qscore** |
| --- | --- | --- | --- |
| **GIST193** | FF | 83.6 | 33.3 |
|  | FFPE | 84.7 | 33.6 |
|  | PB | 76.7 | 31.4 |
| **GIST165** | FF | 84.0 | 33.4 |
|  | FFPE | 85.0 | 33.7 |
|  | PB | 83.4 | 33.3 |
| **GIST174** | FF | 84.4 | 33.6 |
|  | FFPE | 85.9 | 34.0 |
|  | PB | 77.4 | 31.6 |
| **GIST127** | FF | 84.0 | 33.5 |
|  | FFPE | 82.0 | 32.8 |
|  | PB | 78.3 | 31.9 |

**Table S5.** List of somatic somatic disease-related mutations (non-synonimous and nonsense SNVs and InDels located in the coding region and splicing sites). Variants are flagged as shared if present both in FF and FFPE samples, FP if called only in FFPE and FN if called only in FF. Ref: reference; Alt: alternative; Cov: Coverage.

| **GIST174** | | | |  |  |  |  |  |  |  |  |  |  |  |  |  |  |  |  |  |  |
| --- | --- | --- | --- | --- | --- | --- | --- | --- | --- | --- | --- | --- | --- | --- | --- | --- | --- | --- | --- | --- | --- |
| **call** | | **Chr** | **Pos_START** | **Pos_END** | **Ref** | **Alt** | **GENE** | **EXON** | **cDNA** | **PROTEIN** | **FF_Ref_Cov** | **FF_Alt_Cov** | **FF_TOT_Cov** | **FF_Ratio** | **FFPE_Ref_Cov** | **FFPE_Alt_Cov** | **FFPE_TOT_Cov** | **FFPE_Ratio** | **PB_Ref_Cov** | **PB_Alt_Cov** | **PB_TOT_Cov** |
| shared | | 1 | 156907073 | 156907073 | C | G | ARHGEF11 | exon39 | c.G4408C | p.E1470Q | 24 | 13 | 37 | 0.35 | 43 | 22 | 65 | 0.34 | 36 | 0 | 36 |
| shared | | 3 | 100105790 | 100105790 | A | T | TOMM70A | exon2 | c.T357A | p.N119K | 5 | 37 | 42 | 0.88 | 14 | 43 | 57 | 0.75 | 44 | 0 | 44 |
| **shared** | | **4** | **55593661** | **55593661** | **T** | **C** | **KIT** | **exon11** | **c.T1727C** | **p.L576P** | **2** | **160** | **162** | **0.99** | **12** | **236** | **248** | **0.95** | **116** | **0** | **116** |
| shared | | 5 | 147498002 | 147498002 | C | A | SPINK5 | exon23 | c.C2115A | p.D705E | 84 | 39 | 123 | 0.32 | 144 | 67 | 211 | 0.32 | 103 | 0 | 103 |
| shared | | 6 | 7229440 | 7229440 | C | T | RREB1 | exon10 | c.C1108T | p.Q370X | 12 | 6 | 18 | 0.33 | 18 | 6 | 24 | 0.25 | 20 | 0 | 20 |
| shared | | 7 | 137773485 | 137773485 | C | T | AKR1D1 | exon2 | c.C232T | p.R78W | 138 | 32 | 170 | 0.19 | 161 | 44 | 205 | 0.21 | 79 | 0 | 79 |
| shared | | 7 | 6730595 | 6730595 | G | A | ZNF12 | exon6 | c.C1864T | p.P622S | 25 | 55 | 80 | 0.69 | 38 | 68 | 106 | 0.64 | 48 | 0 | 48 |
| shared | | 7 | 86416099 | 86416099 | C | T | GRM3 | exon3 | c.C991T | p.R331C | 27 | 55 | 82 | 0.67 | 21 | 61 | 82 | 0.74 | 50 | 0 | 50 |
| shared | | 9 | 95080946 | 95080946 | C | 0 | NOL8 | exon6 | c.440delG | p.G147fs | 38 | 23 | 61 | 0.38 | 55 | 17 | 72 | 0.24 | 47 | 0 | 47 |
| shared | | 9 | 107267402 | 107267402 | C | T | OR13F1 | exon1 | c.C859T | p.P287S | 43 | 16 | 59 | 0.27 | 51 | 20 | 71 | 0.28 | 41 | 0 | 41 |
| shared | | 9 | 72874151 | 72874151 | A | G | SMC5 | exon1 | c.A157G | p.I53V | 0 | 4 | 4 | 1.00 | 1 | 16 | 17 | 0.94 | 20 | 0 | 20 |
| shared | | 10 | 89717672 | 89717672 | C | T | PTEN | exon7 | c.C697T | p.R233X | 4 | 86 | 90 | 0.96 | 10 | 82 | 92 | 0.89 | 104 | 0 | 104 |
| shared | | 11 | 59626654 | 59626654 | T | C | TCN1 | exon5 | c.A643G | p.K215E | 65 | 58 | 123 | 0.47 | 87 | 52 | 139 | 0.37 | 128 | 0 | 128 |
| shared | | 11 | 4929468 | 4929468 | T | G | OR51A7 | exon1 | c.T869G | p.V290G | 39 | 44 | 83 | 0.53 | 72 | 53 | 125 | 0.42 | 58 | 0 | 58 |
| shared | | 11 | 76062335 | 76062335 | G | A | PRKRIR | exon5 | c.C1859T | p.S620L | 18 | 7 | 25 | 0.28 | 12 | 10 | 22 | 0.45 | 22 | 0 | 22 |
| shared | | 12 | 31613306 | 31613306 | C | T | DENND5B | exon4 | c.G914A | p.R305H | 15 | 14 | 29 | 0.48 | 17 | 13 | 30 | 0.43 | 20 | 0 | 20 |
| shared | | 14 | 24573054 | 24573054 | T | C | PCK2 | exon10 | c.T1804C | p.F602L | 74 | 39 | 113 | 0.35 | 90 | 47 | 137 | 0.34 | 107 | 0 | 107 |
| shared | | 15 | 82575077 | 82575077 | G | A | FAM154B | exon3 | c.G871A | p.A291T | 22 | 19 | 41 | 0.46 | 35 | 31 | 66 | 0.47 | 39 | 0 | 39 |
| shared | | 16 | 23417425 | 23417425 | C | T | COG7 | exon12 | c.G1634A | p.S545N | 69 | 45 | 114 | 0.39 | 56 | 48 | 104 | 0.46 | 92 | 0 | 92 |
| shared | | 17 | 39464308 | 39464308 | 0 | A | KRTAP16-1 | exon1 | c.1198_1199insT | p.I400fs | 28 | 25 | 53 | 0.47 | 40 | 19 | 59 | 0.32 | 38 | 0 | 38 |
| shared | | 19 | 54677857 | 54677857 | G | A | MBOAT7 | exon6 | c.C1081T | p.H361Y | 8 | 4 | 12 | 0.33 | 7 | 4 | 11 | 0.36 | 12 | 0 | 12 |
| shared | | 19 | 54632438 | 54632438 | G | C | PRPF31 | exon12 | c.G1153C | p.E385Q | 63 | 55 | 118 | 0.47 | 77 | 48 | 125 | 0.38 | 111 | 0 | 111 |
| shared | | 22 | 32194614 | 32194614 | C | A | DEPDC5 | exon13 | c.C918A | p.Y306X | 10 | 18 | 28 | 0.64 | 6 | 22 | 28 | 0.79 | 37 | 0 | 37 |
| shared | | X | 2835863 | 2835863 | G | T | ARSD | exon5 | c.C845A | p.A282D | 9 | 2 | 11 | 0.18 | 9 | 4 | 13 | 0.31 | 8 | 0 | 8 |
| FP | | 1 | 31898633 | 31898633 | C | A | SERINC2 | exon6 | c.C510A | p.Y170X | 17 | 0 | 17 | 0.00 | 17 | 5 | 22 | 0.23 | 22 | 0 | 22 |
| FN | | 17 | 7414906 | 7414906 | C | A | POLR2A | exon24 | c.C4100A | p.T1367K | 4 | 2 | 6 | 0.33 | 12 | 0 | 12 | 0.00 | 11 | 0 | 11 |
| FN | | 22 | 50689330 | 50689330 | C | A | HDAC10 | exon2 | c.G64T | p.E22X | 8 | 4 | 12 | 0.33 | 23 | 0 | 23 | 0.00 | 14 | 0 | 14 |
| FN | | 13 | 21549467 | 21549467 | G | A | LATS2 | exon8 | c.C2809T | p.Q937X | 5 | 11 | 16 | 0.69 | 2 | 1 | 3 | 0.33 | 16 | 0 | 16 |
|  | |  |  |  |  |  |  |  |  |  |  |  |  |  |  |  |  |  |  |  |  |
|  | |  |  |  |  |  |  |  |  |  |  |  |  |  |  |  |  |  |  |  |  |
| **GIST165** | | | |  |  |  |  |  |  |  |  |  |  |  |  |  |  |  |  |  |  |
| **call** | **Chr** | | **Pos_START** | **Pos_END** | **Ref** | **Alt** | **GENE** | **EXON** | **cDNA** | **PROTEIN** | **FF_Ref_Cov** | **FF_Alt_Cov** | **FF_TOT_Cov** | **FF_Ratio** | **FFPE_Ref_Cov** | **FFPE_Alt_Cov** | **FFPE_TOT_Cov** | **FFPE_Ratio** | **PB_Ref_Cov** | **PB_Alt_Cov** | **PB_TOT_Cov** |
| shared | 11 | | 47074006 | 47074006 | C | A | C11orf49 | exon3 | c.C217A | p.H73N | 20 | 6 | 26 | 0.23 | 16 | 11 | 27 | 0.41 | 34 | 0 | 34 |
| shared | 22 | | 36960886 | 36960886 | C | T | CACNG2 | exon4 | c.G484A | p.G162R | 32 | 14 | 46 | 0.30 | 41 | 26 | 67 | 0.39 | 46 | 0 | 46 |
| shared | 12 | | 7651706 | 7651706 | C | T | CD163 | exon4 | c.G536A | p.R179Q | 25 | 14 | 39 | 0.36 | 33 | 12 | 45 | 0.27 | 28 | 0 | 28 |
| shared | 2 | | 63175810 | 63175810 | G | A | EHBP1 | exon12 | c.G1829A | p.R610H | 23 | 7 | 30 | 0.23 | 20 | 20 | 40 | 0.50 | 31 | 0 | 31 |
| shared | 6 | | 82461728 | 82461742 | CCGCCGAAGTCGCCG | 0 | FAM46A | exon2 | c.117_131del | p.39_44del | 15 | 4 | 19 | 0.21 | 13 | 7 | 20 | 0.35 | 10 | 0 | 10 |
| shared | 15 | | 75932050 | 75932050 | T | C | IMP3 | exon1 | c.A460G | p.M154V | 33 | 13 | 46 | 0.28 | 26 | 23 | 49 | 0.47 | 57 | 0 | 57 |
| shared | 4 | | 57181800 | 57181800 | A | T | KIAA1211 | exon8 | c.A2132T | p.K711M | 58 | 23 | 81 | 0.28 | 62 | 20 | 82 | 0.24 | 52 | 0 | 52 |
| shared | 11 | | 4976013 | 4976013 | A | G | OR51A2 | exon1 | c.T931C | p.W311R | 18 | 2 | 20 | 0.10 | 13 | 4 | 17 | 0.24 | 12 | 0 | 12 |
| **shared** | **4** | | **55152093** | **55152093** | **A** | **T** | **PDGFRA** | **exon18** | **c.A2525T** | **p.D842V** | **109** | **35** | **144** | **0.24** | **57** | **32** | **89** | **0.36** | **110** | **0** | **110** |
| shared | 12 | | 93139312 | 93139312 | C | T | PLEKHG7 | exon5 | c.C260T | p.A87V | 27 | 9 | 36 | 0.25 | 34 | 15 | 49 | 0.31 | 41 | 0 | 41 |
| shared | 1 | | 29609216 | 29609216 | C | T | PTPRU | exon12 | c.C1897T | p.R633W | 20 | 8 | 28 | 0.29 | 23 | 11 | 34 | 0.32 | 28 | 0 | 28 |
| shared | 16 | | 31485945 | 31485945 | C | T | TGFB1I1 | exon7 | c.C530T | p.S177F | 12 | 8 | 20 | 0.40 | 11 | 4 | 15 | 0.27 | 16 | 0 | 16 |
| shared | 9 | | 136857331 | 136857331 | C | A | VAV2 | exon1 | c.G70T | p.V24L | 28 | 11 | 39 | 0.28 | 50 | 25 | 75 | 0.33 | 42 | 0 | 42 |
| FP | 16 | | 4310481 | 4310481 | C | T | TFAP4 | exon5 | c.G641A | p.R214Q | 25 | 0 | 25 | 0.00 | 9 | 6 | 15 | 0.40 | 18 | 0 | 18 |
|  |  | |  |  |  |  |  |  |  |  |  |  |  |  |  |  |  |  |  |  |  |
|  |  | |  |  |  |  |  |  |  |  |  |  |  |  |  |  |  |  |  |  |  |
| **GIST193** | | | |  |  |  |  |  |  |  |  |  |  |  |  |  |  |  |  |  |  |
| **call** | **Chr** | | **Pos_START** | **Pos_END** | **Ref** | **Alt** | **GENE** | **EXON** | **cDNA** | **PROTEIN** | **FF_Ref_Cov** | **FF_Alt_Cov** | **FF_TOT_Cov** | **FF_Ratio** | **FFPE_Ref_Cov** | **FFPE_Alt_Cov** | **FFPE_TOT_Cov** | **FFPE_Ratio** | **PB_Ref_Cov** | **PB_Alt_Cov** | **PB_TOT_Cov** |
| shared | 21 | | 40665932 | 40665932 | A | C | BRWD1 | exon8 | c.T636G | p.I212M | 31 | 10 | 41 | 0.24 | 35 | 14 | 49 | 0.29 | 24 | 0 | 24 |
| shared | 22 | | 38477115 | 38477138 | CGCCAGGGCGCCGCACGCCGGGCG | 0 | SLC16A8 | exon4 | c.907_930del | p.303_310del | 24 | 13 | 37 | 0.35 | 43 | 21 | 64 | 0.33 | 22 | 0 | 22 |
| shared | 5 | | 23527637 | 23527637 | A | C | PRDM9 | exon11 | c.A2440C | p.S814R | 30 | 7 | 37 | 0.19 | 34 | 14 | 48 | 0.29 | 40 | 2 | 42 |
| FP | 19 | | 2321808 | 2321808 | C | A | LSM7 | exon4 | c.G183T | p.Q61H | 14 | 0 | 14 | 0.00 | 13 | 5 | 18 | 0.28 | 11 | 0 | 11 |
| FN | 11 | | 134118721 | 134118721 | T | C | THYN1 | exon7 | c.A613G | p.I205V | 52 | 14 | 66 | 0.21 | 58 | 0 | 58 | 0.00 | 34 | 0 | 34 |
|  |  | |  |  |  |  |  |  |  |  |  |  |  |  |  |  |  |  |  |  |  |
|  |  | |  |  |  |  |  |  |  |  |  |  |  |  |  |  |  |  |  |  |  |
| **GIST127** | | | |  |  |  |  |  |  |  |  |  |  |  |  |  |  |  |  |  |  |
| **call** | **Chr** | | **Pos_START** | **Pos_END** | **Ref** | **Alt** | **GENE** | **EXON** | **cDNA** | **PROTEIN** | **FF_Ref_Cov** | **FF_Alt_Cov** | **FF_TOT_Cov** | **FF_Ratio** | **FFPE_Ref_Cov** | **FFPE_Alt_Cov** | **FFPE_TOT_Cov** | **FFPE_Ratio** | **PB_Ref_Cov** | **PB_Alt_Cov** | **PB_TOT_Cov** |
| shared | 10 | | 29783908 | 29783908 | A | G | SVIL | exon18 | c.T2498C | p.M833T | 69 | 3 | 72 | 0.04 | 19 | 10 | 29 | 0.34 | 56 | 1 | 57 |
| shared | 3 | | 32032046 | 32032046 | G | A | ZNF860 | exon2 | c.G1475A | p.R492H | 64 | 4 | 68 | 0.06 | 7 | 6 | 13 | 0.46 | 59 | 0 | 59 |
| shared | 9 | | 77416954 | 77416954 | C | T | TRPM6 | exon16 | c.G1854A | p.M618I | 152 | 63 | 215 | 0.29 | 51 | 12 | 63 | 0.19 | 146 | 0 | 146 |
| shared | 1 | | 6149067 | 6149067 | G | A | KCNAB2 | exon9 | c.372-1G>A | - | 33 | 14 | 47 | 0.30 | 4 | 5 | 9 | 0.56 | 25 | 0 | 25 |
| shared | 1 | | 17982540 | 17982540 | T | C | ARHGEF10L | exon22 | c.T2531C | p.V844A | 36 | 27 | 63 | 0.43 | 14 | 13 | 27 | 0.48 | 57 | 0 | 57 |
| shared | 12 | | 113748080 | 113748080 | A | T | SLC8B1 | exon12 | c.T1216A | p.F406I | 15 | 12 | 27 | 0.44 | 14 | 11 | 25 | 0.44 | 32 | 0 | 32 |
| shared | 12 | | 130927026 | 130927026 | C | A | RIMBP2 | exon8 | c.G820T | p.D274Y | 32 | 26 | 58 | 0.45 | 14 | 3 | 17 | 0.18 | 52 | 0 | 52 |
| shared | 6 | | 43487480 | 43487480 | A | G | POLR1C | exon4 | c.A286G | p.N96D | 77 | 64 | 141 | 0.45 | 10 | 15 | 25 | 0.60 | 118 | 0 | 118 |
| shared | 20 | | 3761856 | 3761856 | G | C | SPEF1 | exon1 | c.C79G | p.R27G | 20 | 17 | 37 | 0.46 | 24 | 9 | 33 | 0.27 | 35 | 0 | 35 |
| shared | 12 | | 52772063 | 52772063 | C | T | KRT84 | exon9 | c.G1558A | p.V520I | 15 | 15 | 30 | 0.50 | 10 | 4 | 14 | 0.29 | 16 | 0 | 16 |
| shared | 6 | | 41707655 | 41707655 | C | A | PGC | exon7 | c.G797T | p.W266L | 9 | 21 | 30 | 0.70 | 2 | 6 | 8 | 0.75 | 22 | 0 | 22 |
| shared | 7 | | 129663500 | 129663500 | C | A | ZC3HC1 | exon9 | c.G1021T | p.D341Y | 6 | 10 | 16 | 0.63 | 0 | 6 | 6 | 1.00 | 24 | 0 | 24 |
| shared | 11 | | 1651645 | 1651645 | A | G | KRTAP5-5 | exon1 | c.A575G | p.Y192C | 11 | 9 | 20 | 0.45 | 3 | 4 | 7 | 0.57 | 19 | 0 | 19 |
| FP | 17 | | 42636503 | 42636503 | G | A | FZD2 | exon1 | c.G1447A | p.E483K | 58 | 0 | 58 | 0.00 | 16 | 4 | 20 | 0.20 | 45 | 1 | 46 |
| FP | 1 | | 27057938 | 27057938 | C | T | ARID1A | exon3 | c.C1646T | p.P549L | 29 | 0 | 29 | 0.00 | 19 | 5 | 24 | 0.21 | 38 | 0 | 38 |
| FP | 20 | | 3759409 | 3759409 | C | T | SPEF1 | exon5 | c.G463A | p.G155R | 70 | 0 | 70 | 0.00 | 15 | 4 | 19 | 0.21 | 58 | 0 | 58 |
| FP | 5 | | 176011929 | 176011929 | G | A | CDHR2 | exon19 | c.G2647A | p.D883N | 39 | 0 | 39 | 0.00 | 15 | 4 | 19 | 0.21 | 37 | 0 | 37 |
| FP | 5 | | 176084557 | 176084557 | C | T | TSPAN17 | exon9 | c.C848T | p.T283M | 72 | 0 | 72 | 0.00 | 14 | 4 | 18 | 0.22 | 39 | 0 | 39 |
| FP | 4 | | 106156594 | 106156594 | C | A | TET2 | exon3 | c.C1495A | p.P499T | 58 | 0 | 58 | 0.00 | 14 | 4 | 18 | 0.22 | 46 | 0 | 46 |
| FP | 1 | | 156818752 | 156818752 | G | A | INSRR | exon7 | c.C1532T | p.A511V | 23 | 0 | 23 | 0.00 | 14 | 4 | 18 | 0.22 | 22 | 0 | 22 |
| FP | 17 | | 48447412 | 48447412 | C | T | MRPL27 | exon3 | c.G221A | p.R74H | 55 | 0 | 55 | 0.00 | 14 | 4 | 18 | 0.22 | 63 | 0 | 63 |
| FP | 12 | | 7922711 | 7922711 | G | C | NANOGNB | exon2 | c.G235C | p.E79Q | 74 | 0 | 74 | 0.00 | 17 | 5 | 22 | 0.23 | 52 | 0 | 52 |
| FP | 1 | | 109837826 | 109837826 | G | A | MYBPHL | exon7 | c.C961T | p.R321C | 34 | 0 | 34 | 0.00 | 13 | 4 | 17 | 0.24 | 59 | 0 | 59 |
| FP | 19 | | 45404315 | 45404315 | G | T | TOMM40 | exon8 | c.G797T | p.G266V | 18 | 0 | 18 | 0.00 | 13 | 4 | 17 | 0.24 | 49 | 0 | 49 |
| FP | 21 | | 34166564 | 34166564 | C | A | C21orf62 | exon2 | c.G169T | p.A57S | 65 | 0 | 65 | 0.00 | 16 | 5 | 21 | 0.24 | 63 | 0 | 63 |
| FP | 1 | | 229730771 | 229730771 | G | A | TAF5L | exon5 | c.C1043T | p.T348M | 20 | 0 | 20 | 0.00 | 12 | 4 | 16 | 0.25 | 21 | 0 | 21 |
| FP | 7 | | 142627197 | 142627197 | G | A | TRPV5 | exon3 | c.C305T | p.A102V | 62 | 0 | 62 | 0.00 | 17 | 6 | 23 | 0.26 | 80 | 0 | 80 |
| FP | 14 | | 102901142 | 102901142 | G | T | TECPR2 | exon9 | c.G1988T | p.W663L | 34 | 0 | 34 | 0.00 | 10 | 4 | 14 | 0.29 | 33 | 0 | 33 |
| FP | 2 | | 105858922 | 105858922 | G | A | GPR45 | exon1 | c.G607A | p.V203M | 84 | 0 | 84 | 0.00 | 44 | 18 | 62 | 0.29 | 58 | 0 | 58 |
| FP | 15 | | 84659942 | 84659942 | G | A | ADAMTSL3 | exon23 | c.G3949A | p.V1317M | 49 | 0 | 49 | 0.00 | 8 | 4 | 12 | 0.33 | 62 | 0 | 62 |
| FP | 12 | | 6838600 | 6838600 | G | A | COPS7A | exon5 | c.G515A | p.R172Q | 34 | 0 | 34 | 0.00 | 8 | 4 | 12 | 0.33 | 25 | 0 | 25 |
| FP | 17 | | 8422203 | 8422203 | C | T | MYH10 | exon20 | c.G2339A | p.R780Q | 33 | 0 | 33 | 0.00 | 7 | 4 | 11 | 0.36 | 20 | 0 | 20 |
| FP | 1 | | 43830303 | 43830303 | G | A | ELOVL1 | exon6 | c.C391T | p.R131X | 42 | 0 | 42 | 0.00 | 3 | 4 | 7 | 0.57 | 30 | 0 | 30 |
| FN | 6 | | 49666154 | 49666154 | C | A | CRISP2 | exon6 | c.G338T | p.S113I | 36 | 19 | 55 | 0.35 | 17 | 0 | 17 | 0.00 | 36 | 0 | 36 |
| FN | 5 | | 75896757 | 75896757 | G | A | IQGAP2 | exon10 | c.G1042A | p.A348T | 28 | 18 | 46 | 0.39 | 11 | 0 | 11 | 0.00 | 25 | 0 | 25 |
| FN | 12 | | 110234385 | 110234385 | G | A | TRPV4 | exon5 | c.C1136T | p.T379M | 54 | 14 | 68 | 0.21 | 53 | 0 | 53 | 0.00 | 44 | 0 | 44 |
| FN | 12 | | 11546686 | 11546686 | C | T | PRB2 | exon3 | c.G326A | p.R109Q | 18 | 5 | 23 | 0.22 | 2 | 0 | 2 | 0.00 | 7 | 0 | 7 |
| FN | 19 | | 55263898 | 55263898 | G | A | KIR2DL3 | exon8 | c.G953A | p.R318H | 14 | 4 | 18 | 0.22 | 6 | 0 | 6 | 0.00 | 38 | 0 | 38 |
| FN | 22 | | 31479241 | 31479241 | C | A | SMTN | exon2 | c.C159A | p.S53R | 10 | 4 | 14 | 0.29 | 0 | 0 | 0 | 0.00 | 15 | 0 | 15 |
| FN | X | | 153069154 | 153069154 | T | G | PDZD4 | exon8 | c.A1964C | p.E655A | 18 | 12 | 30 | 0.40 | 3 | 1 | 4 | 0.25 | 25 | 0 | 25 |
| FN | 5 | | 79854548 | 79854548 | C | T | ANKRD34B | exon5 | c.G1291A | p.G431S | 17 | 15 | 32 | 0.47 | 5 | 0 | 5 | 0.00 | 50 | 0 | 50 |
| FN | 19 | | 3964961 | 3964961 | G | A | DAPK3 | exon2 | c.C91T | p.R31W | 8 | 9 | 17 | 0.53 | 0 | 1 | 1 | 1.00 | 8 | 0 | 8 |
| FN | 5 | | 10564638 | 10564638 | C | T | ANKRD33B | exon1 | c.C59T | p.P20L | 15 | 29 | 44 | 0.66 | 0 | 2 | 2 | 1.00 | 21 | 0 | 21 |
| FN | 8 | | 144808844 | 144808844 | G | 0 | FAM83H | exon5 | c.2787delC | p.P929fs | 9 | 19 | 28 | 0.68 | 0 | 2 | 2 | 1.00 | 12 | 0 | 12 |
| FN | 22 | | 37771346 | 37771346 | G | A | ELFN2 | exon3 | c.C229T | p.R77C | 3 | 14 | 17 | 0.82 | 0 | 0 | 0 | 0.00 | 16 | 0 | 16 |
| FN | 21 | | 46935658 | 46935658 | G | A | SLC19A1 | exon5 | c.C1570T | p.P524S | 37 | 22 | 59 | 0.37 | 5 | 0 | 5 | 0.00 | 47 | 0 | 47 |

**Figure S1**. Tagmented DNA library size distribution as analyzed by Agilent DNA 1000 kit.

**Figure S2.** Average insert size of the exome libraries sequenced at 100bp x 2.

**Figure S3.** Percentage of reads mapping on-target and off-target, i.e. outside the 37Mb Nextera target region used for exome enrichment.
